# Monocyte TLR2/4 as predictive biomarkers for progression from dengue warning signs to severe disease

**DOI:** 10.3389/fimmu.2026.1901740

**Published:** 2026-08-19

**Authors:** Shiqian Chen, Peng Ye, Peiao Rao, Tiantian Sun, Jiali Qi, Ying Zheng, Xiao-Qiang Yu, Fan Zou, Xihua Fu, Ruonan Zhang

**Affiliations:** 1 Infection Medicine Research Institute of Panyu District, Affiliated Panyu Central Hospital, Guangzhou Medical University, Guangzhou, Guangdong, China; 2 Guangdong Provincial Key Laboratory of Insect Developmental Biology and Applied Technology, Guangzhou Key Laboratory of Insect Development Regulation and Application Research, Institute of Insect Science and Technology, School of Life Sciences, South China Normal University, Guangzhou, China; 3 Faculty of Pharmaceutical Sciences, Shenzhen University of Advanced Technology, Shenzhen, China

**Keywords:** biomarker, dengue, monocyte, severity stratification, toll-like receptor

## Abstract

**Background:**

The World Health Organization (WHO) classification system, comprising dengue fever (DF), dengue with warning signs (DWS), and severe dengue (SD), has been widely adopted globally; however, clinical management remains challenging. Current warning sign criteria lack specificity, frequently failing to distinguish patients progressing to severe disease from those with transient illness, thereby contributing to delayed interventions and missed therapeutic windows. Although Toll-like receptors (TLRs) mediate innate antiviral immunity and are implicated in severe dengue pathogenesis, their expression dynamics during the critical DWS transition window, a phase in which intervention could prevent progression to severe disease, remain entirely unknown.

**Methods:**

An integrated biomarker discovery study was conducted, comprising bioinformatics analysis of publicly available single-cell RNA sequencing (scRNA-seq) data (GSE220969) and experimental validation in an independent clinical cohort of 45 dengue patients and 10 healthy controls (H) in Guangdong Province, China. Phase-specific immune signatures were identified from the scRNA-seq data. TLRs expression was quantified by multiparameter flow cytometry. Diagnostic performance was assessed using ROC curve analysis, and correlations with clinical laboratory parameters were evaluated using Spearman’s rank correlation.

**Results:**

Bioinformatics analysis of 23 cell clusters revealed that TLR2/4/7/8 expression peaked specifically during the DWS phase, predominantly in monocytes. Flow cytometry confirmed elevated surface TLR2 (AUC 0.874) and TLR4 (AUC 0.763) in DWS versus other phases, with discriminatory capacity to distinguish DWS from SD (TLR2: AUC 0.833; TLR4: AUC 0.810). Moreover, correlation analysis of TLRs expression with clinical indicators demonstrated that the expression levels of TLR2 and TLR4 were associated with the development of SD.

**Conclusions:**

Elevated monocyte TLR2/4 expression during DWS identifies patients at risk for progression to severe dengue before clinical decompensation. These mechanism-informed biomarkers provide a foundation for point-of-care risk stratification in endemic regions.

## Background

Dengue virus (DENV) is the most common mosquito-borne viral infection globally, causing an estimated 390 million infections annually, of which 96 million are symptomatic (1). Americas, Southeast Asia, and the Western Pacific bear the greatest burden, with expanding transmission in previously non-endemic regions driven by climate change, urbanization, and global travel (2, 3). In 2024, the World Health Organization (WHO) reported over 14.6 million global dengue cases and > 12,000 deaths, with the Americas alone accounting for > 13 million cases (4). China, particularly Guangdong and Yunnan Provinces, has experienced resurgent endemic transmission, with Guangdong reporting sustained local outbreaks in 2023-2024 (5, 6).

According to the WHO 2009 guidelines, dengue is classified into three clinical stages: dengue fever (DF), dengue with warning signs (DWS), and severe dengue (SD) (7). However, this framework exhibits significant diagnostic ambiguity in practice. The boundary between DF and DWS is often unclear, and the transition from DWS to SD lacks quantifiable predictive indicators, leaving clinicians in a dilemma between overtreatment and undertreatment. For instance, a 2023–2024 prospective cohort study in Colombia revealed that among 600 virologically confirmed patients, 28% developed severe clinical manifestations, yet only 22 met WHO 2009 SD criteria while 145 were classified as DWS with more severe disease courses (8). This discrepancy highlights the inability of current classification to capture intermediate states. Consequently, the molecular mechanisms underlying disease progression remain incompletely elucidated, and existing clinical indicators, such as liver and kidney function markers, exhibit limited discriminatory capacity (9, 10).

The DWS phase represents a critical clinical juncture where appropriate intervention can prevent progression to life-threatening complications. Although patients with warning signs are recommended for hospitalization, current criteria demonstrate limited specificity (9, 10), as most patients do not progress to SD, resulting in unnecessary admissions that increase healthcare burden in endemic regions (8, 11). Conversely, atypical presentations without classical warning signs, such as symptoms resembling enteric fever or malaria, can progress rapidly to severe disease (11–15). This diagnostic ambiguity complicates clinical decision-making and may delay intervention in patients at genuine risk. These limitations highlight the need for objective biomarkers capable of identifying DWS patients at genuine risk of progression to SD.

The innate immune response, particularly monocyte activation, is central to DENV pathogenesis (16–18). Monocytes, dendritic cells, and macrophages are primary target cells for DENV and central to immune activation (19–21). These cells recognize pathogen-associated molecular patterns (PAMPs) through pattern recognition receptors (PRRs), among which Toll-like receptors (TLRs) are particularly important for detecting viral components and initiating antiviral responses (20, 22, 23). TLR2 recognizes DENV envelope protein and non-structural protein 1 (NS1), initiating downstream signaling that facilitates viral clearance (24). However, TLRs activation also contributes to immunopathology: DENV infection upregulates TLR2 and TLR4, driving excessive inflammation linked to plasma leakage and hemorrhagic events (25–27), while DENV-induced TLR3 signaling exacerbates vascular dysfunction (23, 28, 29).

Previous studies have documented TLRs upregulation in severe dengue (23, 25–27), establishing these receptors as markers of established pathology. Whether TLRs can similarly serve as predictive biomarkers during the DWS phase, the critical interval for preventive intervention, remains entirely unexplored. Specifically, whether TLRs exhibit differential expression during DWS compared to both DF and SD remains unknown.

To bridge this gap, we employed a two-stage integrative approach combining computational analysis with experimental validation. First, we conducted bioinformatics analysis of publicly available single-cell RNA sequencing (scRNA-seq) datasets to characterize immune cell dynamics and TLR pathway activation across dengue severity phases. This discovery-phase analysis identified upregulation of *TLR1*, *TLR2*, *TLR4*, *TLR7*, and *TLR8* specifically during DWS, predominantly within monocytes. Second, we validated these findings using flow cytometry analysis of an independent clinical cohort from Guangdong Province, China. This validation-phase study confirmed that surface TLR2 and TLR4 expression on peripheral monocytes exhibits high predictive accuracy for disease progression and correlates with clinical severity indicators including thrombocytopenia. This dual-phase strategy establishes monocyte TLR2/4 as predictive biomarkers for dengue severity stratification during the critical warning sign phase.

## Materials and methods

### Analysis of single-cell RNA-sequencing data

Publicly available scRNA-seq data (NCBI GEO Accession: GSE220969) were analyzed using the Seurat R package. Quality control was performed with DoubletFinder to remove doublets. Cells with fewer than 400 detected features, total UMI counts < 1,000 or > 30,000, or mitochondrial gene content > 15% were excluded. Genes detected in fewer than three cells were also removed.

Batch effects were corrected using the Harmony package. Gene expression was normalized using the “LogNormalize” method in the NormalizeData function. The top 2,000 highly variable genes were identified using the “vst” method in FindVariableFeatures. Z-score normalization was applied using ScaleData. Dimensionality reduction was conducted via principal component analysis (PCA), and the first 20 principal components were selected for subsequent analysis. Cell clusters were manually annotated based on canonical marker genes with a clustering resolution set to 0.3 (30). Differentially expressed genes (DEGs) were identified using the FindMarkers function, with an adjusted p-value < 0.05 considered statistically significant (31). Kyoto Encyclopedia of Genes and Genomes (KEGG) pathway enrichment analysis was conducted using the enrichKEGG function. Pathways with a q-value < 0.05 were considered significantly enriched. Pathway activity across different groups was assessed using Gene Set Variation Analysis (GSVA). Heatmaps were generated using the pheatmap package, with data normalized by Z-score.

Correlations between TLRs and clinical indicators were calculated using Pearson correlation analysis. Receiver operating characteristic (ROC) curves for TLR2, TLR4, TLR7, and TLR8 were plotted using the pROC package, and the area under the curve (AUC) was calculated.

### Clinical data collection

Demographic characteristics, clinical manifestations, and routine laboratory parameters were retrospectively collected from the hospital’s electronic medical record system. Blood samples for routine laboratory testing and Peripheral blood mononuclear cell (PBMC) isolation were collected simultaneously from each participant at enrollment, prior to any therapeutic intervention.

### Subject selection

45 dengue patients were enrolled in 2024 from the Affiliated Panyu Central Hospital of Guangzhou Medical University. Dengue infection was confirmed by NS1 antigen rapid test or serological detection of dengue-specific antibodies. Patients were classified into DF, DWS, and SD groups according to WHO 2009 guidelines.

Inclusion criteria were: age ≥ 18 years; positive for dengue IgM and/or NS1; good compliance and informed consent. Exclusion criteria included: comorbidities such as hepatitis, cirrhosis, cancer, or other thrombocytopenic disorders; history of hematologic malignancy, bleeding disorders, chronic liver disease, or diabetic nephropathy; or other conditions deemed unsuitable by investigators.

Healthy controls (H) Group Criteria: No history of DF; No history of fever or other acute illnesses within the past 3 months; No chronic diseases (e.g., diabetes, hepatitis, heart disease) or active infections.

### PBMC isolation

A total of 4–5 mL of whole blood was collected in EDTA-anticoagulated tubes. Red blood cells (RBCs) lysis buffer was added at a 1:5 (blood:lysis buffer) ratio, followed by gentle pipetting to mix thoroughly. After incubation at room temperature for 5 minutes, the sample was centrifuged at 500×g for 5 minutes at 4 °C, and the supernatant was discarded. The cell pellet was resuspended in 4 mL of phosphate-buffered saline (PBS), gently mixed, and centrifuged again at 450×g for 5 minutes at 4 °C. The reddish supernatant (containing residual lysed RBCs) was carefully aspirated. The resulting cell pellet was resuspended in freezing medium, transferred to cryovials, stored at –80 °C for 24 hours, and subsequently transferred to liquid nitrogen for long-term preservation.

### Flow cytometry

**Figure 1:**
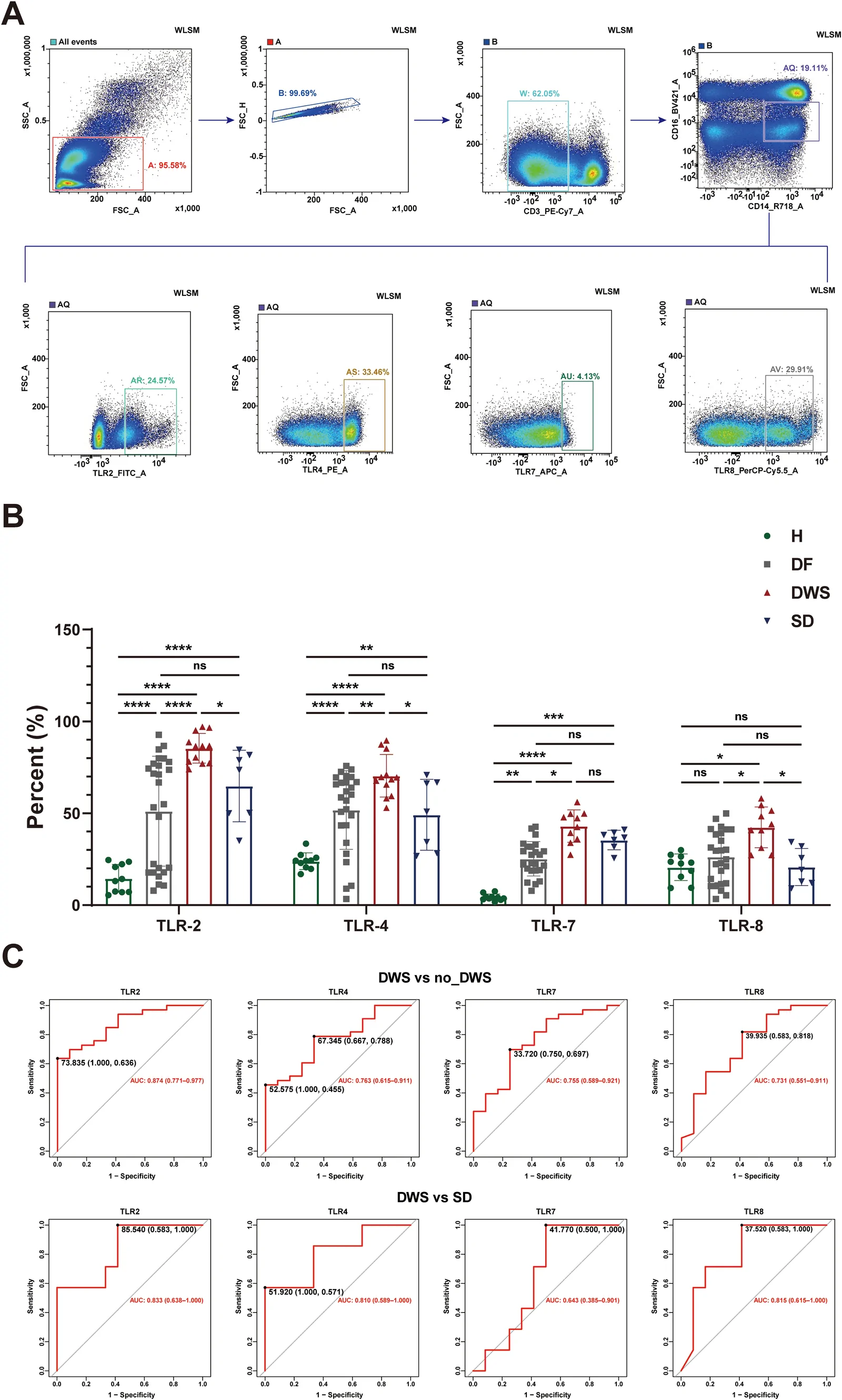
Flow cytometry validation of TLR2/4 as predictive biomarkers for the SD progression. **(A)** Representative gating strategy. Following exclusion of doublets and dead cells, CD3^-^ lymphocytes were excluded. Monocytes were identified as CD14^+^ cells. TLRs expression levels were subsequently assessed within the gated monocyte population. **(B)** TLR2, TLR4, TLR7, and TLR8 expression levels across groups. Each point represents an individual participant. Significance determined by two-way ANOVA: *p < 0.05, **p < 0.01, ***p < 0.001, ****p < 0.0001. **(C)** ROC curves for TLRs in distinguishing DWS from non-DWS groups (top) and DWS from SD (bottom). AUC values indicate diagnostic performance. H (n = 10), DF (n = 26), DWS (n = 12), SD (n = 7).

Cryopreserved PBMCs were rapidly thawed in a 37 °C water bath, washed with pre-warmed RPMI-1640 medium, and resuspended in PBS containing 1% BSA. After centrifugation at 200×g for 5 minutes at 4 °C, cells were incubated with fluorochrome-conjugated antibodies against human CD3, CD14, CD16, TLR2, TLR4, TLR7, and TLR8 (detailed in **Supplementary Table 1**) for 30 minutes at 4 °C in the dark. Cells were then fixed in 1% paraformaldehyde for 24 hours at 4°C. Data acquisition was performed on an Aurora spectral flow cytometer (Cytek Biosciences), and analysis was conducted using FlowJo software (version 10.9, Tree Star). Gating strategies are illustrated in **Figure 1A**.

## Result

### scRNA-seq reveals dynamic immune cell composition alterations across dengue severity phases

**Figure 2:**
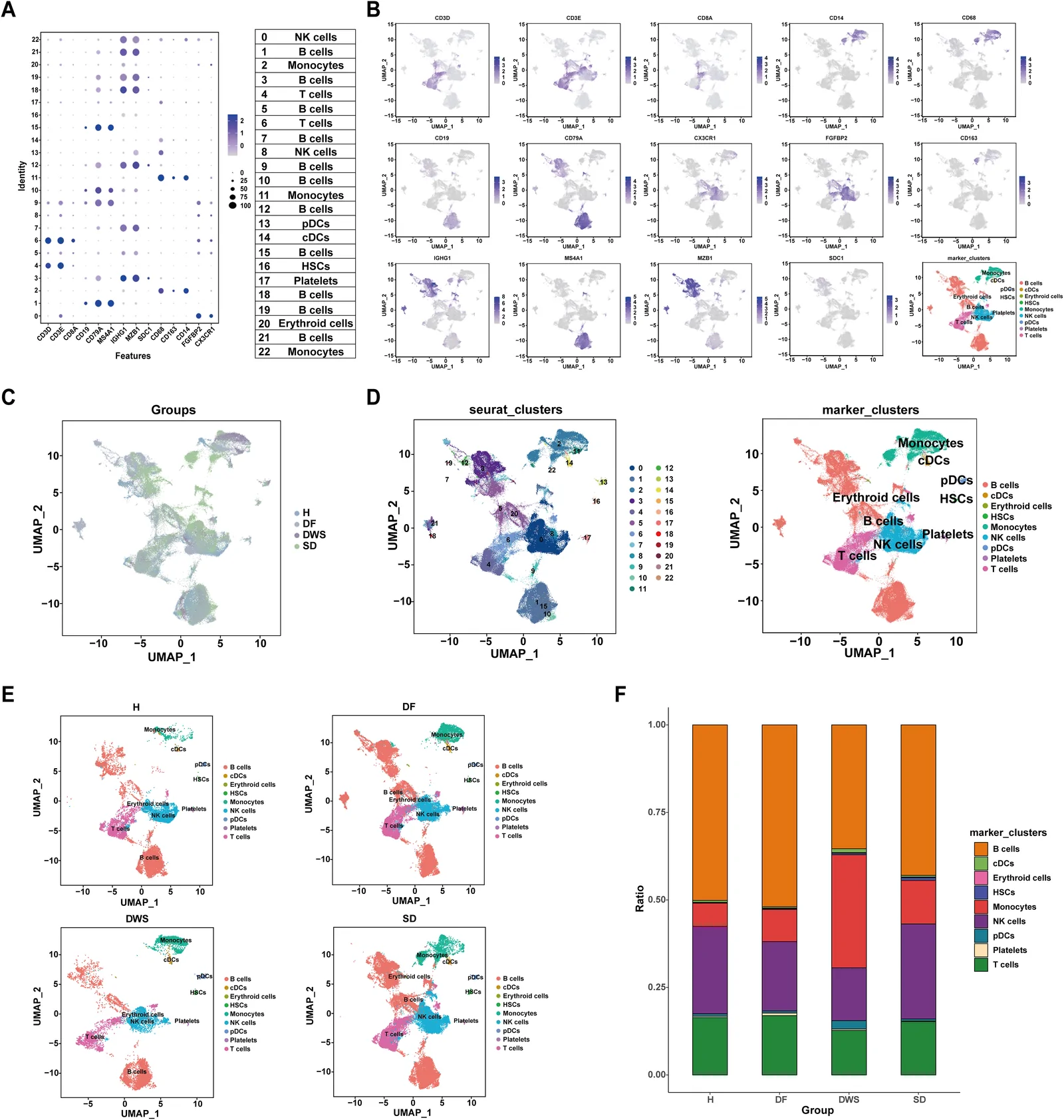
ScRNA-seq reveals dynamic alterations in immune cell composition across dengue severity phases. **(A)** Bubble plot depicting marker gene expression for 23 single-cell clusters (left) and cell type annotations (right). **(B)** UMAP projection showing marker gene expression for major cell populations. **(C)** UMAP visualization by clinical group. **(D)** UMAP visualization by cluster (left) and cell type (right). **(E)** Separate UMAP projections for each group. **(F)** Stacked bar chart showing cell type proportions across groups. UMAP, uniform manifold approximation and projection.

To characterize PBMC heterogeneity in DENV infection, publicly available scRNA-seq data (GSE220969) were analyzed. Using established lineage-specific marker genes (**Figure 2A**), 23 transcriptionally distinct clusters were identified and annotated into nine major cell types: B cells, conventional dendritic cells (cDCs), erythroid cells, hematopoietic stem cells (HSCs), monocytes, natural killer (NK) cells, plasmacytoid dendritic cells (pDCs), platelets, and T cells (**Figure 2B**).

Comparative analysis revealed increased monocyte relative abundance across all dengue-infected groups compared to H (**Figures 2C–F**). Notably, the DWS group exhibited the most pronounced monocyte expansion relative to lymphocyte populations, as NK cells, T cells, and B cells decreased. This shift in abundance resulted in monocytes representing the largest PBMC fraction in DWS.

### TLR pathway activation peaks in monocytes during the DWS phase

**Figure 3:**
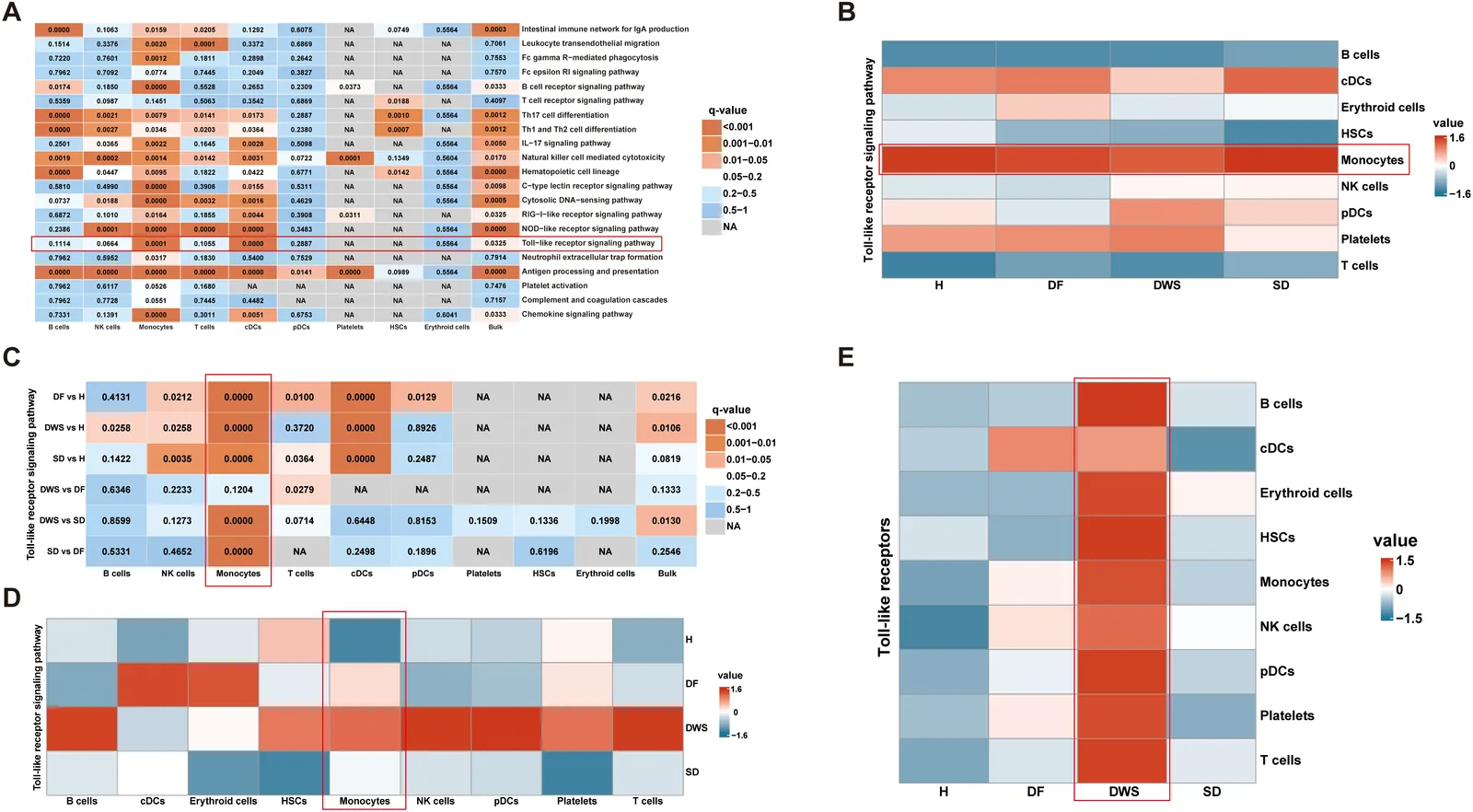
TLR signaling pathway activation peaks in monocytes during the DWS phase. **(A)** Heatmap depicting 21 immune-related pathways enriched by DEGs across cell types. Values represent the q-value. **(B)** Heatmap showing TLR signaling pathway expression within groups across cell types. The color scale represents Z-score normalized expression values. **(C)** Heatmap illustrating between-group expression differences of the TLR signaling pathways within the same cell type. Values represent the q-value. **(D)** Heatmap displaying TLR signaling pathway expression within the cell types across groups. The color scale represents Z-score normalized expression values. **(E)** Heatmap of *TLRs* expression levels within cell types across groups. The color scale represents Z-score normalized expression values. Red boxes highlight DWS-specific upregulation of the TLR pathway or *TLRs* in monocytes.

To systematically assess immune pathway dynamics, differential analysis of 21 immune-related pathways was performed across all cell types between dengue patients and H. Significant enrichment of TLR signaling pathway genes was observed specifically within monocytes and cDCs (**Figure 3A**). Pathway enrichment analysis further confirmed that the TLR signaling pathway exhibited the strongest enrichment in monocytes across all four groups (**Figure 3B**). Inter-group comparative analysis revealed that TLR pathway enrichment in monocytes was statistically significant across all disease transitions, including DF versus H (q < 0.001), DWS versus H (q < 0.001), SD versus H (q < 0.001), and notably DWS versus SD (q < 0.001), indicating sustained pathway activation throughout disease progression with particular relevance to the DWS transition window (**Figure 3C**).

Disease stage-specific analysis revealed that monocyte TLR signaling peaked specifically during the DWS phase, with activity scores markedly elevated compared to H, DF, and SD phases (**Figure 3D**). This temporal pattern was corroborated at the individual receptor level: gene expression analysis revealed that multiple *TLRs* (*TLR1*, *TLR2*, *TLR4*, *TLR7*, and *TLR8*) were coordinately upregulated in DWS, predominantly within monocytes, whereas expression levels were substantially lower in both DF and SD phases (**Figure 3E**). Collectively, these data establish monocyte-centric TLR signaling as a temporally dynamic and phase-specific hallmark of the DWS phase.

### Altered monocyte subset composition modulates pro-inflammatory effector output during DWS

**Figure 4:**
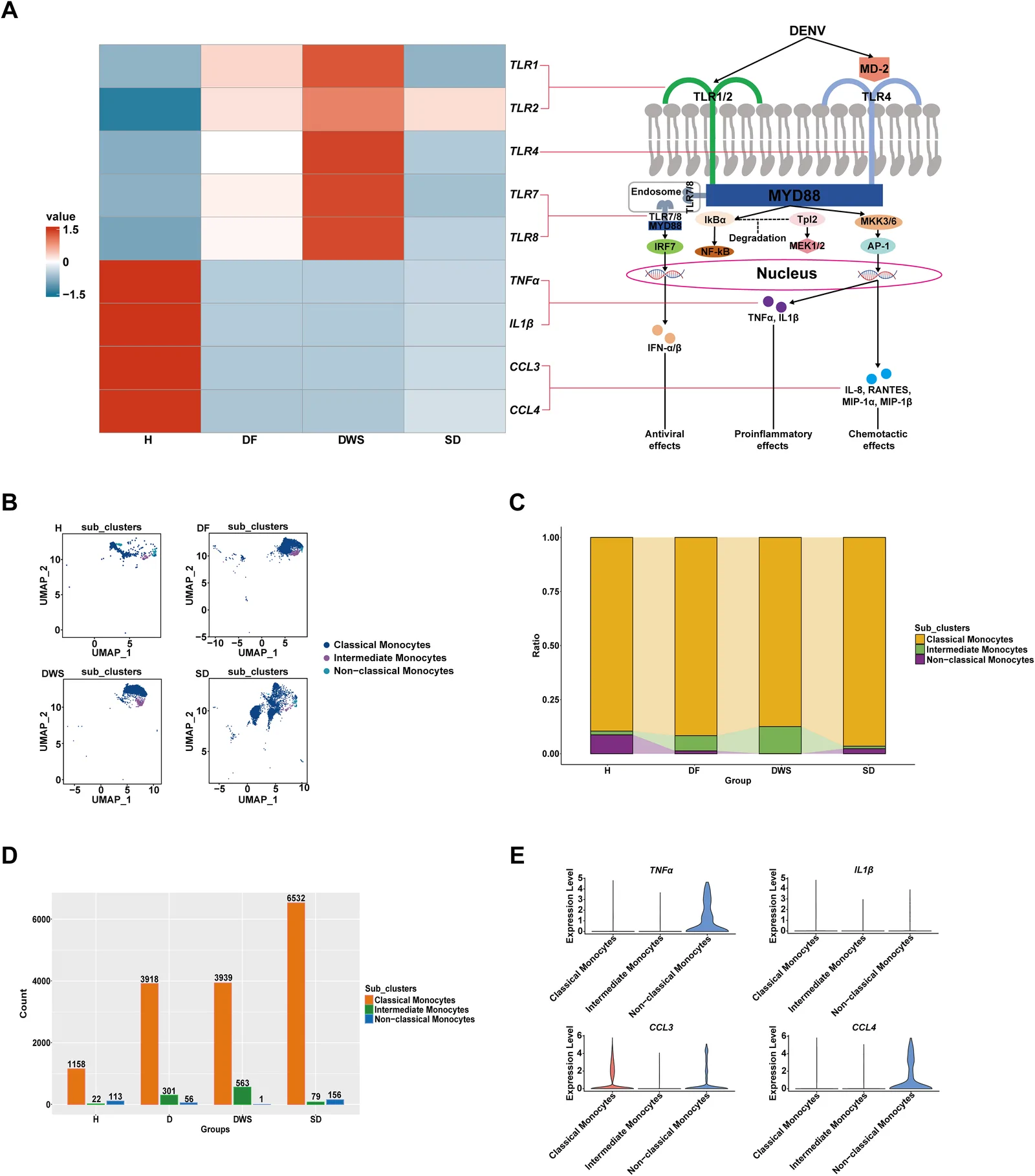
Altered monocyte subset composition modulates pro-inflammatory effector output during DWS. **(A)** Heatmap showing expression of *TLRs* (*TLR1*, *TLR2*, *TLR4*, *TLR7*, and *TLR8*) and downstream cytokines in monocytes, with schematic diagram of TLR signaling cascade. **(B)** UMAP plots of monocyte subsets across groups. **(C)** Stacked bar chart showing proportional abundance of monocyte subsets. **(D)** Bar graphs showing the absolute cell counts of monocyte subsets across groups. **(E)** Violin plots depicting expression of *TNFα*, *IL1β*, *CCL3*, and *CCL4* in CMs, IMs, and NMs. UMAP, uniform manifold approximation and projection.

Building upon the observed monocyte TLR signature, analysis of TLR signaling-related genes revealed differential expression of downstream effectors. Despite high expression of multiple *TLRs* (*TLR1*, *TLR2*, *TLR4*, *TLR7*, and *TLR8*) in DWS, key pro-inflammatory cytokines including *TNF α*, *IL 1β*, *CCL4* and *CCL3* were downregulated (**Figure 4A**), indicating a dissociation between receptor signaling and effector output.

To mechanistically explain this dissociation, monocyte subset composition was investigated. Consistent with established monocyte subset classification (32, 33), single-cell transcriptomic reanalysis identified three canonical subpopulations: classical monocytes (CMs; CD14^+^^+^CD16^-^), intermediate monocytes (IMs; CD14^+^^+^CD16^+^), and non-classical monocytes (NMs; CD14^+^CD16^+^^+^) (**Figure 4B**). Proportional analysis revealed that NMs abundance was lowest in DWS (**Figure 4C**). NMs are established primary producers of pro-inflammatory cytokines (33, 34). Absolute cell quantification was performed to assess whether this decreased abundance reflected cellular loss or dilution. This analysis demonstrated NMs depletion in DWS, with the lowest absolute counts among all disease phases (**Figure 4D**). Gene expression analysis showed that NMs had the highest expression of *TNFα* and *CCL4*, with *CCL3* also enriched in this subset (**Figure 4E**). *IL1β* expression was minimal across all subsets. These findings are consistent with NMs depletion contributing to reduced inflammatory output in DWS.

### Clinical cohort validation of TLR expression dynamics in dengue progression

To validate the phase-specific TLRs signature identified in scRNA-seq analysis, multiparameter flow cytometry was performed in an independent clinical cohort. A total of 10 healthy volunteers and 45 dengue patients with varying disease severity were enrolled. According to WHO 2009 classification criteria, patients were categorized as DF (n = 26), DWS (n = 12), and SD (n = 7) (**Table 1**). Clinical parameters including hematocrit (HCT), platelet count, albumin, and hepatic transaminases (aspartate aminotransferase [AST] and alanine aminotransferase [ALT]) were assessed. DWS patients exhibited significantly reduced platelet counts compared to both DF and SD patients. Both DWS and SD patients demonstrated mild elevations in AST and ALT relative to DF, while HCT and albumin showed no significant differences between groups (**Supplementary Figure S1**). Detailed demographic and clinical characteristics are provided in **Supplementary Table 2**.

**Table 1:** Statistical table of clinical characteristics of DENV patients.

Clinical characteristics	DF (Dengue fever)	DWS (Dengue with warning signs)	SD (Severe dengue)
Number of subjects	26	12	7
Fever	26	10	6
Rash	4	4	1
Pain	25	8	5
Nausea, Diarrhea	1	2	2
Abdominal pain, Persistent vomiting	0	1	1
Clinical fluid accumulation	0	2	3
Lethargy, Restlessness	0	1	1
impaired consciousness	0	0	1
Shock (<90/60mmHg)	0	0	3
Mucosal bleeding	0	3	0
Severe bleeding	0	0	3
Severe organ damage	0	0	2
Thrombocytopenia (<50×10^9^/L)	0	9	1
platelet count (mean ± SD, 10^9^/L)	131 ± 58.18	49.58 ± 39.25	98.83 ± 48.9
HCT (mean ± SD, %)	39.97 ± 5.64	39.23 ± 6.16	35.74 ± 9.64
AST (mean ± SD, U/L)	51.41 ± 24.13	79 ± 38.05	117 ± 90.83
ALT (mean ± SD, U/L)	26.55 ± 12.1	42.67 ± 19.5	57.4 ± 46.16
Albumin (mean ± SD, g/L)	38.48 ± 2.63	36.33 ± 2.88	35.17 ± 3.35

HCT, Hematocrit; AST, Aspartate Aminotransferase; ALT, Alanine Aminotransferase. For detailed clinical characteristics of enrolled patients, please refer to the **Supplementary Table 2**.

PBMCs were isolated from all participants, and monocytes were identified by sequential gating of CD3^-^and CD14^+^(**Figure 1A**), and TLRs expression was quantified. TLR2, TLR4, TLR7, and TLR8 expression was elevated in DWS compared to H, D and DF groups (**Figure 1B**, **Supplementary Table 3**). ROC analysis demonstrated discrimination of DWS from non-DWS groups for all four TLRs (TLR2: AUC 0.874; TLR4: AUC 0.763; TLR7: AUC 0.755; TLR8: AUC 0.731). For the critical DWS versus SD comparison, TLR2 (AUC 0.833), TLR4 (AUC 0.810), and TLR8 (AUC 0.815) maintained performance, whereas TLR7 failed to maintain consistent discriminatory capacity between DWS and SD, with portions of its ROC curve falling below the diagonal (**Figure 1C**). These findings identified TLR2, TLR4, and TLR8 as candidate biomarkers with diagnostic potential for SD progression.

Given that dengue progression in vulnerable populations such as elderly individuals and those with comorbidities may present with more atypical or accelerated clinical features, we therefore analyzed whether TLR2/4 expression levels differ among these subgroups. No significant differences were observed across clinical stages in either elderly (> 60 years) or comorbid patients (**Supplementary Figure S2A**), Furthermore, correlation analysis between age and TLR2/4 expression showed no strong correlation in DWS patients (r = 0.045 and r = 0.023), but the expression levels remained consistently elevated, suggesting that high TLR2/TLR4 expression is a relatively stable pathological hallmark of DWS, independent of age, in this adult cohort (**Supplementary Figure S2B**).

### TLR2 and TLR4 as mechanism-informed biomarkers for organ-specific dysfunction

**Figure 5:**
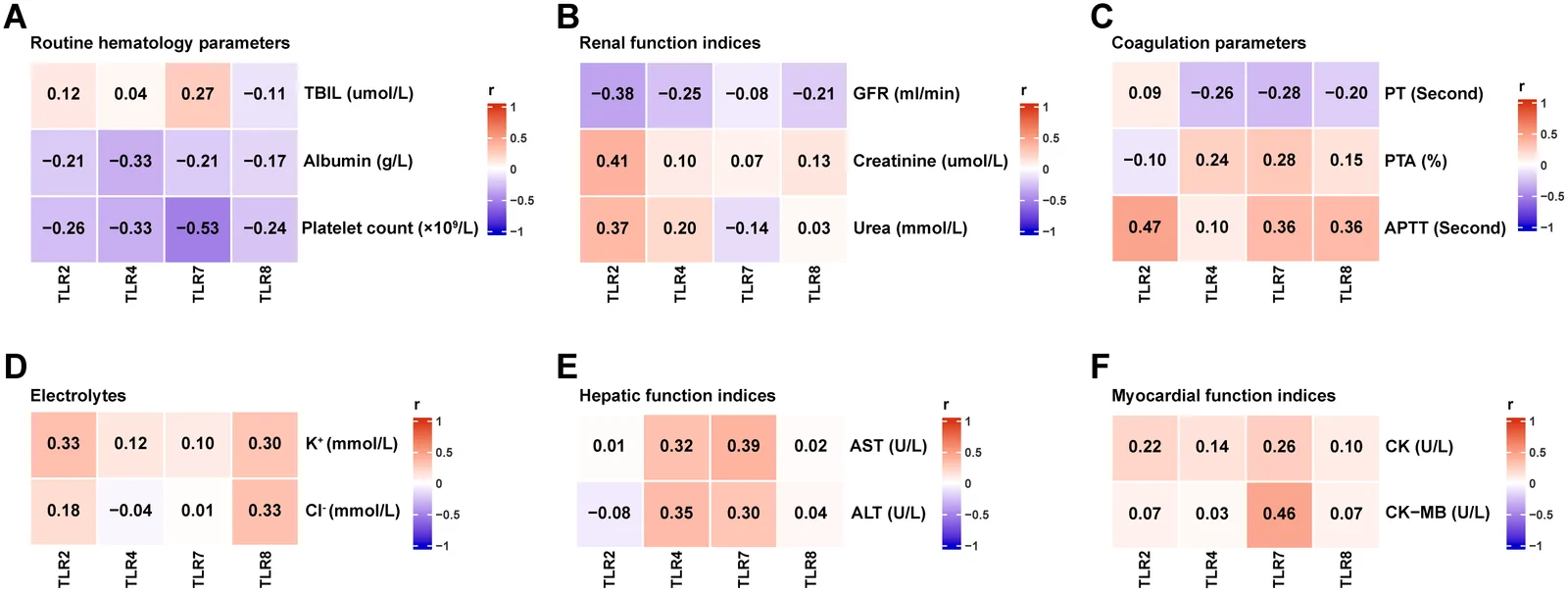
Clinical correlation analysis identifies TLR2/4 associations with organ-specific dysfunction indicators. **(A)**Pearson correlation analysis of TLR2, TLR4, TLR7, and TLR8 expression levels with **(A)** routine hematology parameters, **(B)** renal function indices, **(C)** coagulation parameters, **(D)** electrolytes, **(E)** hepatic function indices, and **(F)** myocardial function indices. Color intensity indicates correlation strength (red, positive; blue, negative). Key correlations: TLR2/4 with platelet count (r = -0.26 to -0.33), TLR2 with creatinine (r = 0.41), urea (r = 0.37), and APTT (r = 0.47), TLR4 with ALT (r = 0.35), AST (r = 0.32), and albumin (r = -0.33), and TLR7 with platelet count (r = -0.53).

To determine the clinical relevance of candidate TLR biomarkers, correlation analysis was performed with disease severity indicators (**Figures 5A–F**). TLR2 expression was inversely associated with platelet count (r = −0.26), with elevated TLR2 marking impending thrombocytopenia, alongside positive correlations with renal function markers (creatinine, r = 0.41; urea, r = 0.37) and coagulation parameters (activated partial thromboplastin time [APTT], r = 0.47). TLR4 showed a similar inverse correlation with platelet count (r = −0.33) and positive correlations with hepatic transaminases (AST, r = 0.32; ALT, r = 0.35) and negative correlation with albumin (r = −0.33). These inverse associations indicate that higher TLR2/4 expression predicts lower platelet counts, identifying patients at risk for hemorrhagic complications before severe thrombocytopenia manifests. TLR8 exhibited weaker correlations with clinical parameters. Notably, TLR2 and TLR4 demonstrated the strongest and most distinct clinical associations, marking renal/coagulation dysfunction and hepatic injury/plasma leakage risks, respectively. These mechanism-specific correlation patterns support the selection of TLR2 and TLR4 as prioritized biomarkers for early risk stratification in dengue.

## Discussion

TLRs have long been recognized as sentinels of innate immunity against DENV infection (23, 35, 36). While prior studies documented TLRs upregulation in severe dengue, establishing them as markers of end-stage pathology (26, 27, 37–41), a key question remains unresolved: whether TLRs can predict progression to severe disease before the manifestation of life-threatening complications.

Our integrative approach, combining bioinformatics reanalysis of publicly available scRNA-seq data with clinical flow cytometry validation in an independent clinical cohort, demonstrates that monocyte-specific TLR2 and TLR4 expression peaks uniquely during DWS phase, establishing these molecules as candidate early-warning biomarkers. The discriminatory capacity of these receptors to distinguish DWS from SD (AUC > 0.8) underscores their potential clinical utility in this critical disease transition window.

Although monocytes are recognized as pivotal in DENV pathogenesis (21, 42, 43), precise temporal characterization of their activation has been lacking. The present findings not only confirm significant monocyte expansion and pronounced TLR pathway activation in DWS patients, but through single-cell resolution analysis, identify this activation as a temporally restricted signature specific to the DWS phase, clearly demarcating it from both DF and SD. This observation suggests that TLR signaling in monocytes represents a hallmark of the critical interval wherein immune responses are intensified but have not yet progressed to irreversible severe pathology.

A key and paradoxical finding was the observed downregulation of classic pro-inflammatory effectors *TNFα* and *IL1β* despite high *TLRs* expression during DWS. The single-cell data reanalysis provide a multi-faceted explanation for this complex immune state. Through the lens of monocyte subset dynamics, we identified a significant reduction in NMs, the primary producers of inflammatory cytokines such as TNF-α (33, 34, 44). The depletion of this key effector subset, both in relative abundance and absolute cell numbers, likely contributes to the net reduction in inflammatory output. The mechanism underlying NMs reduction may involve their patrolling function; NMs bind to endothelial cells via CX3CL1 and are potentially sequestered or activated at the vascular endothelium, contributing to increased vascular permeability and their concomitant depletion from circulation (44–46). This observation bridges innate immune activation to the hallmark vascular pathology of SD.

The expression patterns of these TLRs are closely associated with critical clinical and pathological alterations. Correlation analysis revealed that TLR2 and TLR4 expression was linked to markers of renal injury, hepatic injury/plasma leakage, and coagulation dysfunction (**Figure 5**), suggesting that active TLR signaling serves as an early indicator of multi-organ injury risk. Of particular importance, the expression of TLR2 and TLR4 showed significant correlations with thrombocytopenia—a key warning sign predictive of shock and severe hemorrhage (7, 47–51). Existing evidence directly supports the role of TLRs in this process, as the DENV non-structural protein NS1 can activate platelets and trigger hemorrhage via TLR4 (37, 52, 53), while TLR2-mediated viral sensing has been directly correlated with disease pathogenesis (26, 43, 54). Therefore, we propose that the monocyte TLR2/4 signal, which peaks during the DWS phase, is not an epiphenomenon but may constitute a key pathogenic axis that mediates viral sensing on one hand and drives the severe pathological processes characterized by coagulopathy and vascular leakage on the other. Changes in coagulation parameters (prothrombin time [PT], prothrombin activity [PTA], APTT) further suggest that TLR signaling may simultaneously induce a compensatory hypercoagulable state (55), suggesting its complex role in coagulation imbalance.

In clinical practice, integrating monocyte TLRs expression levels with established diagnostic criteria may serve as a valuable reference for disease monitoring and risk stratification in dengue fever. Specifically, a distinctive peak in TLRs expression in DWS patients indicates an elevated risk of disease progression. Conversely, declining TLRs expression in the absence of clinical improvement suggests that the patient may have entered the critical transition phase from DWS to SD. This integrated approach not only enhances the accuracy of clinical diagnosis but also holds promise for guiding earlier targeted interventions, thereby reducing the incidence and mortality of severe dengue and alleviating the overall burden on healthcare systems.

Our study has several limitations. First, the modest SD cohort size (n = 7) limited statistical power. While TLR2/4 expression appeared stable across adult age groups in this cohort (**Supplementary Figure S2**), the absence of pediatric samples and the small subgroup sizes preclude definitive conclusions about age-specific biomarker performance. Validation in larger, multi-center studies is warranted to confirm the generalizability of these findings. Second, both DENV serotype and infection history (primary versus secondary) are important variables in dengue pathogenesis. Differential serotype virulence and antibody-dependent enhancement (ADE) during heterologous secondary infection are well-established determinants of disease severity (56–59). Since serotyping was not performed in our study cohort, we were unable to assess the potential confounding effects of different serotypes or primary/secondary infection status on TLR2/TLR4 expression levels. This limitation is particularly relevant for endemic regions with multiple circulating serotypes. Future studies incorporating serotype stratification are warranted to confirm the generalizability of the TLR2/4 biomarker signature across diverse epidemiological settings. Third, the cross-sectional design comparing independent patient groups (DF, DWS, SD) enabled phase-specific biomarker identification but did not capture within-patient temporal dynamics. Prospective longitudinal studies with serial sampling throughout the clinical progression are essential next steps to confirm the dynamic trajectory of these biomarkers and support their clinical implementation. Finally, association rather than causation is demonstrated; functional studies employing TLR2/4 blockade in DENV-infected monocytes are needed to confirm direct effects on cytokine production, endothelial integrity, and platelet activation. Additionally, evaluation of downstream signaling molecule phosphorylation (e.g., NF-κB, p65, IRF3) would complement our transcriptional findings.

In conclusion, monocyte TLR2/TLR4 expression peaks specifically during the DWS phase and exhibits discriminatory capacity for disease progression. These findings establish a basis for point-of-care risk stratification in dengue-endemic regions. TLR2/4 biomarkers are intended to complement, rather than replace, WHO criteria, enhancing early risk identification during the critical DWS window to enable timely clinical interventions and reduce severe disease incidence.

## Data Availability

Publicly available datasets were analyzed in this study. This data can be found here: GSE220969.

## Supplementary material

Supplementary Figure 1 Clinical laboratory parameters across dengue severity groups. Comparison of **(A)** platelet count, **(B)** HCT, **(C)** albumin, **(D)** ALT, and **(E)** AST levels among DF (n = 26), DWS (n = 12), and SD (n = 7) patients. Each data point represents an individual participant, color-coded by disease severity: DF (green), DWS (red), SD (blue). Statistical significance determined by t-tests or non-parametric tests: *p < 0.05, **p < 0.01, ***p < 0.001; ns, not significant. For detailed clinical characteristics, please refer to the **Supplementary Table 2**.

Supplementary Figure 2 The potential predictive value of TLR2/4 in specific populations (elderly patients and those with comorbidities. **(A)** TLR2 and TLR4 expression levels were compared between elderly (age > 60 years, n = 13) and non-elderly patients (n = 32), as well as between patients with (n = 11) and without comorbidities (n = 34), across clinical stages (DF, DWS, SD). Significance determined by two-way ANOVA: ns, not significant. **(B)** Pearson correlation of age with TLR2 and TLR4 expression in DF (n = 26), DWS (n = 12), and SD (n = 7). R values are shown for each group.

Supplementary Table 1 Antibody list.

Supplementary Table 2 Patients ID and clinical data.

Supplementary Table 3 Statistical analysis of flow cytometry results in H, DF, DWS and SD.
